# A rare case of an extra-oral plasmablastic lymphoma presenting through a scrotal abscess in a 42-year-old man

**DOI:** 10.1308/rcsann.2022.0166

**Published:** 2023-04-13

**Authors:** P Śluzar, A Reekhaye, F MacAskill, M Ong, V Rose, T Yap

**Affiliations:** ^1^ King’s College London, UK; ^2^ Guy’s and St Thomas’ NHS Foundation Trust, UK

**Keywords:** Lymphoma, Plasmablastic, Fournier’s gangrene

## Abstract

Plasmablastic lymphoma (PBL) is a rare lymphoid neoplasm frequently presenting in the oral cavity. It is an aggressive type of non-Hodgkin’s lymphoma that shares pathological features with plasma cell myeloma. In addition to human immunodeficiency virus (HIV), it is also associated with Epstein–Bar virus (EBV) and immunosuppression in HIV-negative patients, for example, post transplantation. Extra-oral PBL is rare and only a few case reports involving the testis have been described. Here we describe the first reported case of PBL presenting with a scrotal abscess (not involving the testes) in a patient newly diagnosed with HIV. This case highlights the rare presentation of a rare disease, the difficulties in establishing a diagnosis and the importance of a timely multidisciplinary approach to its management.

## Case history

A 42-year-old male presented to the emergency department with a 3-week history of pruritus scroti and a rapidly enlarging abscess of the left hemi-scrotum. The patient was apyrexial and denied any night sweats or unintentional weight loss. Prior to this he was referred to the head and neck clinic with a left parotid swelling which had been biopsied. An inflammatory cause was suspected and he was awaiting further review by the infectious diseases team to rule out tuberculosis.

**Figure 1:**
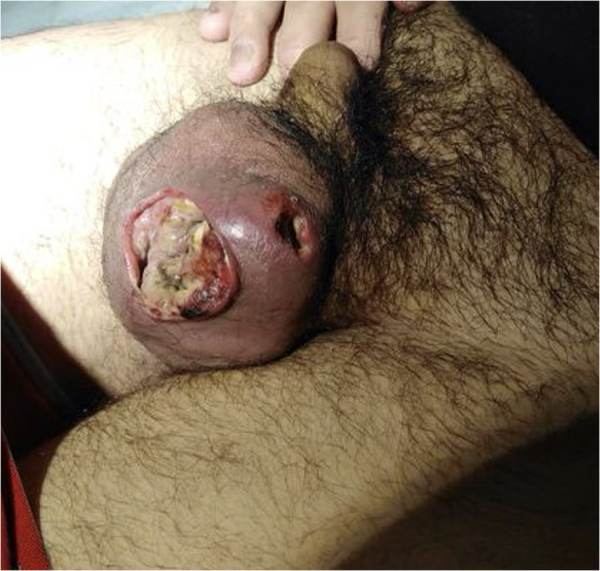
Initial presentation

Examination of his external genitalia revealed a heavily inflamed left hemi-scrotum with two sites of skin ulceration and a malodorous purulent discharge (Figure 1). Initial blood tests showed a normal white cell count and a C-reactive protein of 19mg/l. Owing to the initial suspicion of Fournier’s gangrene (FG), a magnetic resonance imaging (MRI) scan of the pelvis was arranged prior to surgical debridement. A human immunodeficiency virus (HIV) test was also performed on the advice of the microbiologist owing to sexual risk-related factors: homosexuality and recent anal intercourse.

On surgical debridement of the left hemi-scrotum, the ulcerated areas were excised and sent for histopathological and microbiological analysis. The left testis was viable and not involved. The patient was treated with ceftriaxone, metronidazole, doxycycline and co-trimoxazole pending the results.

**Figure 2:**
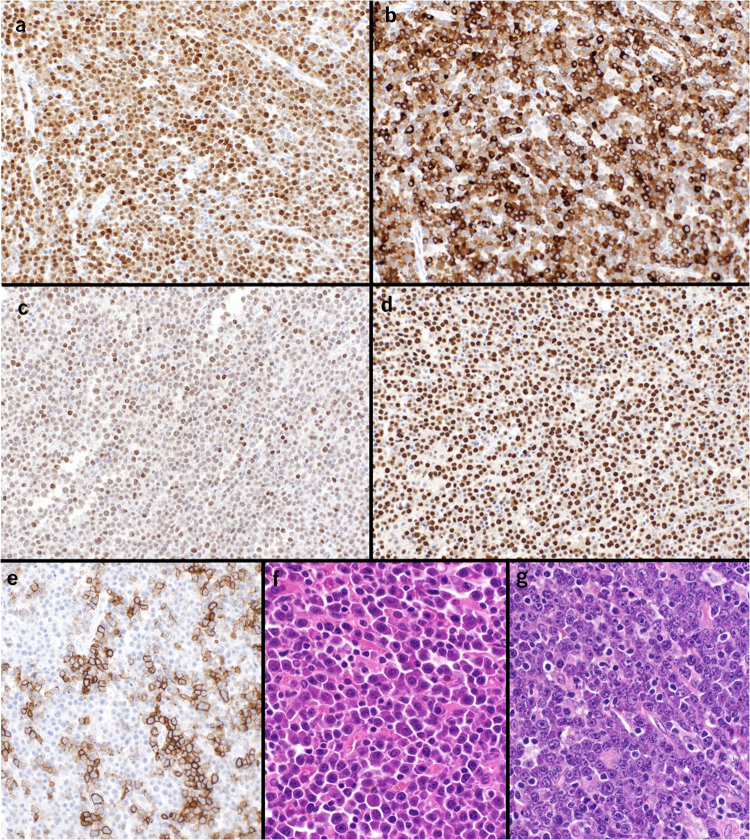
Histopathology: (a) MUM1(+), (b) CD79(+), (c) BLIMP1(+), (d) EBER-ISH(+), (e) CD20(+), original magnification ×20. (f,g) Haematoxylin and eosin stain, original magnification ×40

Biopsy of the lesion eventually showed atypical plasmacytic cells with lambda light chain restriction and a plasmablastic appearance giving the new potential diagnoses of lymphoma or myeloma. The cells were CD45(+), CD79a(+), MUM1(+), BLIMP1(+), CD38(+), CD56(+), Epstein–Bar encoding region in situ hybridisation (EBER-ISH)(+), CD20(+), CD30(−), EMA(−), HHV-8(−) and there was no amyloid deposition (Figure 2). An IGH-MYC rearrangement was identified on fluorescence in situ hybridisation. A bone marrow trephine biopsy showed no evidence of bone marrow involvement by a plasma cell neoplasm or lymphoma. No paraprotein was identified on serum electrophoresis. The diagnosis was therefore more consistent with plasmablastic lymphoma (PBL).

Following two further debridements, a full closure of the scrotal defect with a thigh split skin graft was performed by the plastic surgeons. Primary wound healing allowed for a six-cycle chemotherapy regimen with rituximab, cyclophosphamide, doxorubicin hydrochloride, vincristine and prednisolone (R-CHOP). Post-skin graft, the patient was well and was discharged from the hospital after 22 days.

**Figure 3:**
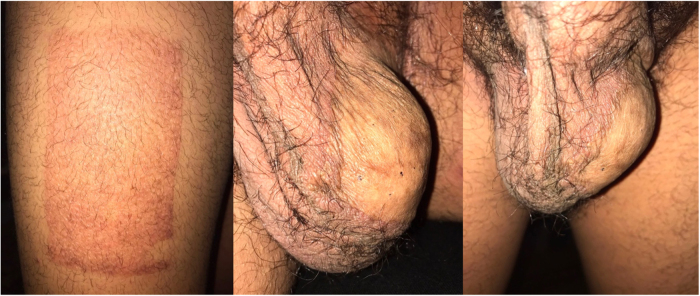
Follow-up at 1 year

Given the new HIV diagnosis, a combined antiretroviral therapy (cART) regimen was initiated and after a month, the viral load had reduced from 119,422 to 4,004copies/ml, CD4 count increased from 73 to 200cells/l and CD4/CD8 improved from 0.11 to 0.23. The patient also underwent two cycles of high-dose methotrexate for prophylaxis against central nervous system relapse. A fluoro-deoxy-glucose positron emission tomography scan 6 months after starting treatment showed complete metabolic response. At 1-year follow-up, the patient reports being fully functional and back to his baseline with PBL in remission (Figure 3).

## Discussion

**Figure 4:**
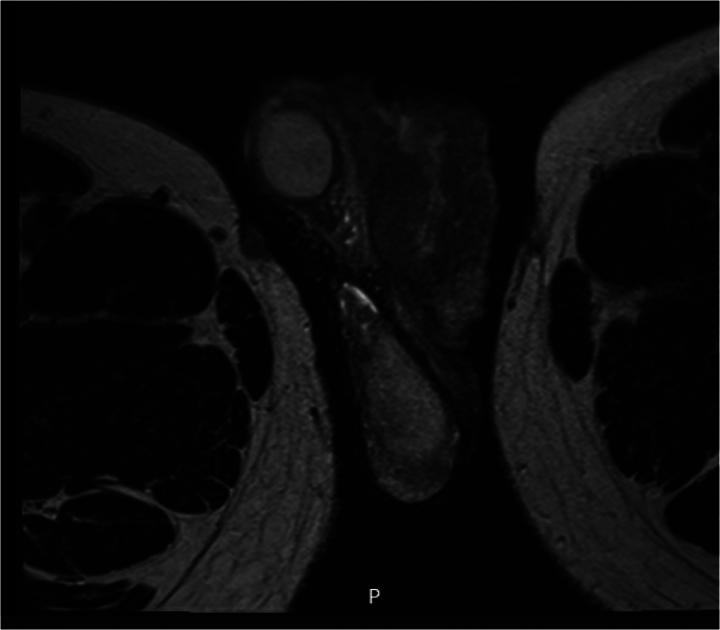
Axial T2-weighted magnetic resonance image

Cutaneous scrotal PBL is a unique presentation of a rare and aggressive lymphoid neoplasm most frequently affecting patients with a degree of immune impairment.^1,2^ From a urological perspective, a fistulating scrotal abscess always raises the suspicion of necrotising fasciitis. In most FG cases, scrotal pain with signs of sepsis mandates an urgent and aggressive surgical debridement. Atypically, our patient presented with stable observations and a very focal complaint without pain. Furthermore, FG as the first manifestation of HIV is extremely rare. For these reasons, an MRI of the pelvis prior to surgical intervention was used to assess for the depth of invasion and the presence of any other lesions or sites of infection such as the rectum (Figure 4). Initial debridement showed similar cutaneous and subcutaneous involvement in contrast to the usually concealed necrotic changes seen in FG. Expedition of histopathological results allowed for a prompt diagnosis of PBL and early involvement of the haematology team.

In a PBL review by Castillo *et al*,^3^ HIV-positive individuals were found to show significantly higher expression of CD20, CD56 and EBER, all of which were seen in our case. Other differentiation markers may include strong positivity for CD38, CD138, MUM1, Blimp1, XBP1 and MYC with less consistent expression for CD45, CD79a, EMA and CD30 as reported previously.^1,4^ Owing to its rarity, PBL poses a diagnostic challenge and its management is especially complex when associated with a severe insult to the immune system. Our patient presented with a CD4 count of 73cells/l and viral load of 119,422copies/ml, making cART and chemotherapy timing difficult with regards to satisfactory wound healing. Despite no standard of care, most patients with PBL are treated with CHOP and CHOP-like regimens as in this case where CD-20-positivity mandated the addition of rituximab.^5^

## Conclusion

PBL is a rare tumour that is most commonly found in patients with HIV. It usually presents as an oral neoplasm but extra-oral presentations involving the testes have been described. Our case is the first reported case of PBL in a newly diagnosed HIV patient presenting as a cutaneous scrotal lesion mimicking FG. It highlights the diagnostic challenges and the complexity of management of PBL in a severely immunocompromised individual.
